# Mirizzi Syndrome With a Hepatic Duct Confluence Fistula and Stone Migration Into the Left Hepatic Duct: A Case Report

**DOI:** 10.7759/cureus.101865

**Published:** 2026-01-19

**Authors:** Harine Siribaddana, Harsh Kandpal, Florian Grimpen, Ian Goh, Manju D Chandrasegaram

**Affiliations:** 1 General Surgery, The Prince Charles Hospital, Brisbane, AUS; 2 Radiology, The Prince Charles Hospital, Brisbane, AUS; 3 Gastroenterology, Royal Brisbane and Women’s Hospital, Brisbane, AUS

**Keywords:** biliary obstruction, cholecystobiliary fistula, endoscopic retrograde cholangiopancreatography, gallstone migration, hepatic duct confluence, left hepatic duct, magnetic resonance cholangiopancreatography, mirizzi syndrome, subtotal cholecystectomy, variant biliary anatomy

## Abstract

Mirizzi syndrome (MS) is an uncommon complication of cholelithiasis, in which an impacted gallstone causes extrahepatic biliary obstruction via extrinsic duct compression. Advanced disease may progress to cholecystobiliary fistula formation. We report the case of a 75-year-old man with cardiometabolic comorbidities who presented with cholestatic liver biochemistry and imaging findings consistent with chronic calculous cholecystitis. Computed tomography and magnetic resonance cholangiopancreatography revealed a distended gallbladder packed with stones, hilar biliary narrowing, and a variant biliary configuration in which the right posterior sectoral duct drained into the left hepatic duct and the right anterior sectoral duct drained into the common hepatic duct. Endoscopic retrograde cholangiopancreatography (ERCP) enabled the clearance of distal common bile duct stones and placement of biliary stents for decompression, but did not definitively delineate the fistula. During laparoscopic surgery, the gallbladder was densely inflamed and intrahepatic, with a sizeable cholecystobiliary fistula at the hepatic duct confluence and intraductal stones within the confluence and the left hepatic duct. We did not identify previous reports describing this pattern of stone migration in the setting of pre-existing variant biliary anatomy. A laparoscopic fundus-first, fenestrated subtotal cholecystectomy was performed along with stone extraction and controlled external drainage. Postoperatively, hepatic and subphrenic abscesses with retained stones were managed with antibiotics, image-guided drainage, repeat ERCP for stent removal, and delayed laparoscopic washout with stone retrieval. This case highlights the limitations of preoperative imaging in complex fistulating MS and supports multimodal investigation, intraoperative contingency strategies, and planned staged management in high-risk patients.

## Introduction

Mirizzi syndrome (MS) is an uncommon complication of cholelithiasis in which an impacted stone in the gallbladder neck, infundibulum (Hartmann’s pouch), or cystic duct causes extrinsic compression of the common hepatic duct (CHD) or common bile duct (CBD) [1-3]. The reported incidence ranges from approximately 0.3% to 5.7% among patients with cholelithiasis [1,2,4,5]. With ongoing inflammation, deeply impacted stones may cause pressure necrosis and mucosal ulceration, progressing to erosion and contained perforation with cholecystobiliary fistula formation [1-5]. Congenital biliary anatomical variants are common (reported in 18%-23% of cases) and can increase diagnostic complexity and operative risk, particularly when the cystic duct runs closely parallel to the CHD or CBD [1,2]. The Csendes classification stratifies MS from type I (extrinsic compression) to types II-IV (increasing bile duct wall destruction with cholecystobiliary fistula) and type V (associated cholecystoenteric fistula and/or gallstone ileus) [3,5]. Clinically, the presentation is often non-specific (commonly jaundice and right upper quadrant pain) with a typical cholestatic biochemical profile [1,2].

MS can mimic hilar malignancies on clinical assessment and imaging. Chronic cholecystitis is a shared inflammatory risk factor for biliary malignancy, and MS may be associated with mild carbohydrate antigen (CA) 19-9 elevation, further complicating its interpretation [1,5,6]. Prior retrospective analyses have diagnosed gallbladder cancer in 5.3%-28% of patients with MS compared to 1% of patients with gallstone disease [5,7,8].

Ultrasonography and computed tomography (CT) are frequently used as first-line investigations. Magnetic resonance cholangiopancreatography (MRCP) is widely cited as the most accurate non-invasive modality for delineating biliary anatomy and supporting the diagnosis of MS, with a reported sensitivity of 77%-100% [1,2,9,10]. Despite being invasive, endoscopic retrograde cholangiopancreatography (ERCP) is regarded as the preoperative reference standard because it provides definitive cholangiography and enables therapeutic interventions such as stone extraction and biliary stenting [1,11].

Surgery remains the definitive treatment. However, the optimal operative strategy is debated because of the low incidence of MS and markedly distorted anatomy in advanced disease [1,2,4,12,13]. While open surgery has historically been the predominant approach to mitigate the risk of bile duct injury, contemporary series and systematic reviews increasingly describe laparoscopic management in selected patients treated in specialist units [4,13-17].

Advanced fistulating MS with involvement at or above the hepatic duct confluence is rare, and proximal intraductal stone migration has only been sporadically described. To our knowledge, we report the first case of gallstones migrating through a cholecystobiliary fistula at the hepatic duct confluence into the left hepatic duct (LHD). This occurred in the setting of a pre-existing biliary variant in which the right posterior sectoral duct drained into the LHD and the right anterior sectoral duct drained into the CHD. This case illustrates the limitations of multimodal imaging in characterising complex fistulating disease, the influence of variant biliary anatomy on stone distribution and operative risk, and the role of staged management with controlled drainage when immediate biliary reconstruction is not feasible. Written informed consent was obtained from the patient for publication of this case report and accompanying images.

This case was previously presented as a poster presentation at the 2024 International Hepato-Pancreato-Biliary Association (IHPBA) World Congress on May 15-18, 2024.

## Case presentation

A 75-year-old man was referred to the general surgical outpatient clinic with deranged liver function tests and an abnormal upper abdominal ultrasound (USS). His comorbidities included coronary artery bypass surgery and mitral valve annuloplasty three months prior, ischemic cardiomyopathy, complete heart block with atrial fibrillation managed with a pacemaker and a direct oral anticoagulant (apixaban), type 2 diabetes mellitus, prostate cancer, gout, and a previous umbilical hernia repair.

Initial blood tests revealed a cholestatic picture with markedly elevated alkaline phosphatase (ALP) and gamma-glutamyl transferase (GGT) levels, moderate transaminase elevation, and normal bilirubin levels (Table 1).

**Table 1:** Laboratory investigation results at the time of referral to the service and at discharge from hospital postoperatively. *: The patient was receiving regular therapeutic direct oral anticoagulation at the time of both blood tests. WBC: white blood cell count; CRP: C-reactive protein; MCV: mean corpuscular volume; ALP: alkaline phosphatase; GGT: gamma-glutamyl transferase; ALT: alanine transaminase; AST: aspartate aminotransferase; INR: international normalized ratio; APTT: activated partial thromboplastin time

Test (unit of measurement)	Reference range	On referral	At discharge
Hemoglobin (g/L)	120–180	113	140
Hematocrit (%)	35–51	37	42
WBC (× 10^9^/L)	3.5–11.0	10.4	13.4
CRP (mg/L)	<5.0	13	24
Platelet count (× 10^9^/L)	140–400	146	340
MCV (fL)	80–100	96	96
Neutrophils (× 10^9^/L)	2.0–8.0	8.08	10.34
Lymphocytes (× 10^9^/L)	1.0–4.0	1.29	1.75
Monocytes (× 10^9^/L)	0.1–1.0	0.95	0.98
Eosinophils (× 10^9^/L)	<0.4	0.06	0.25
Sodium (mmol/L)	135–145	137	137
Potassium (mmol/L)	3.5–5.2	4.6	4.2
Magnesium (mmol/L)	0.70–1.10	0.68	0.76
Calcium (corrected for albumin) (mmol/L)	2.10–2.60	2.57	2.66
Urea (mmol/L)	2.9–8.2	8.8	7.9
Creatinine (µmol/L)	64–108	99	98
Total protein (g/L)	60–80	60	71
Albumin (g/L)	35–50	29	31
Bilirubin – total (µmol/L)	<20	10	9
Bilirubin – conjugated (µmol/L)	<4	<4	<4
ALP (U/L)	30–110	565	135
GGT (U/L)	<55	1,390	146
ALT (U/L)	<45	268	41
AST (U/L)	<35	198	21
INR	0.9–1.2	1.4	1.7
Prothrombin time (seconds)	9–13	16	20
APTT (seconds)	25–38	33	34

An upper abdominal USS performed four months prior demonstrated slightly prominent intrahepatic ducts, a CBD measuring up to 5 mm, and a nodular liver. The gallbladder appeared full of echogenic material, which was thought to represent either a porcelain gallbladder or a large calculus. At the time of referral, the patient denied having biliary colic, jaundice, or systemic symptoms. Examination revealed a palpable, non-tender gallbladder, which was concerning in the context of deranged liver function and extensive comorbidities.

**Figure 1:**
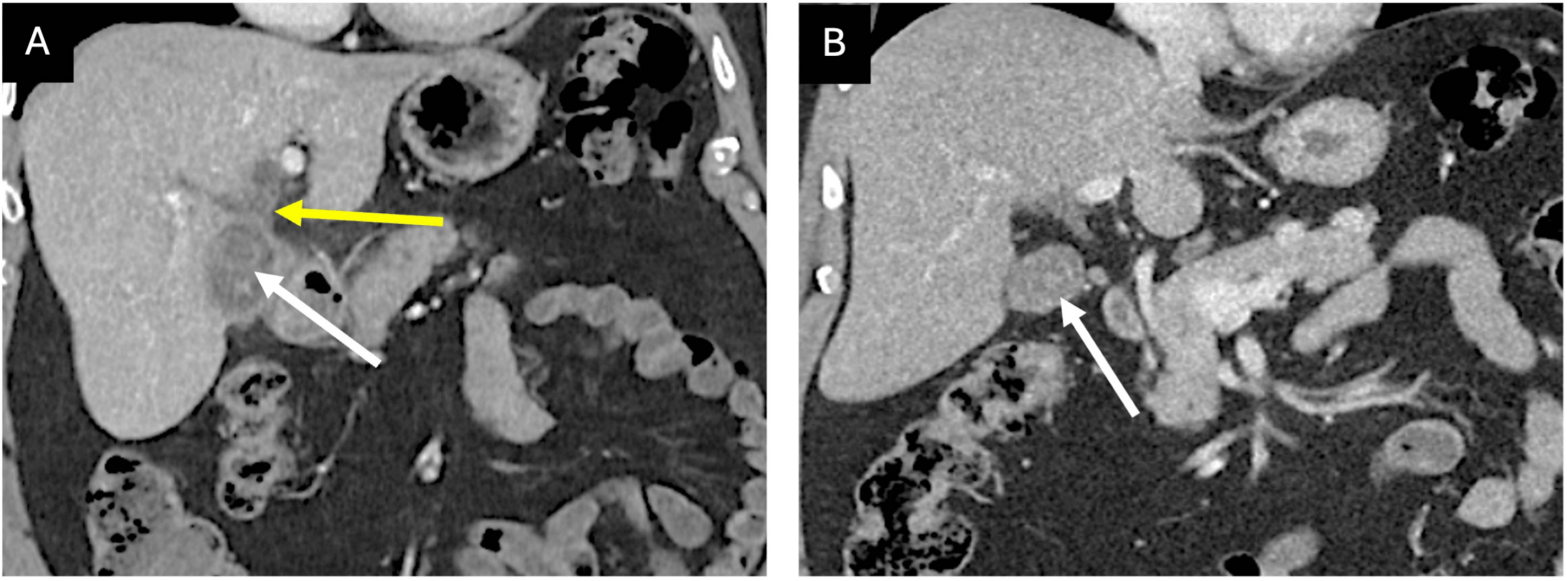
Computed tomography (CT) scan findings at presentation to the outpatient clinic (A) compared to imaging four years prior (B). Portal venous phase CT coronal image (A) demonstrating loss of intervening fat plane between the gallbladder neck and the bile duct in the region of the hepatic duct confluence (yellow arrow). Comparatively, imaging from four years earlier (B) demonstrates a preserved fat plane in the porta between the gallbladder and the common hepatic duct. Gallstones are faintly visible in both scans (white arrows).

A CT scan revealed a thickened, irregular gallbladder wall with an indistinct neck and possible radiolucent stones. The intrahepatic bile ducts were mildly prominent, and there were enlarged lymph nodes at the porta hepatis (Figure 1). These findings were new compared to those of a CT scan performed four years earlier.

**Figure 2:**
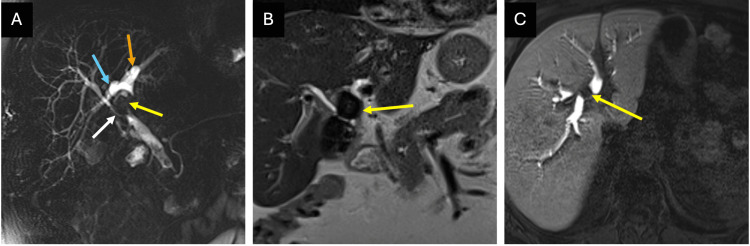
Preoperative magnetic resonance cholangiopancreatography (MRCP) findings. (A) Coronal (projectional) T2-weighted HASTE sequence demonstrates a calculus at the gallbladder neck extruding into the hepatic hilum (yellow arrow) and outlines variant anatomy: the right posterior sectoral duct (blue arrow) drains into the left hepatic duct (orange arrow), while the right anterior sectoral duct drains low into the common hepatic duct (white arrow). Recognition of this variant is important as it may influence stone distribution and increases the complexity of dissection around the confluence. (B) Coronal T2-weighted HASTE sequence demonstrates a calculus (yellow arrow) extending superior to the level of the cystic duct toward the porta hepatis, causing compression of ducts at the hepatic duct confluence. A true intraductal stone position via fistulation was only definitively confirmed at surgery. (C) Axial T1-weighted 3D VIBE sequence after gadoxetate disodium (Primovist) demonstrates a filling defect within the left hepatic duct (yellow arrow). In retrospect, this finding was consistent with intraductal stone migration through a cholecystobiliary fistula at the confluence.

Subsequent MRCP (Figure 2) revealed chronic calculous cholecystitis with a possible previous perforation of the gallbladder. At least two large calculi (up to 25 mm) were identified, extending superior to the level of the cystic duct toward the porta hepatis, causing compression of the hepatic ducts. There was narrowing at the hepatic duct confluence with mild intrahepatic duct dilatation and non-obstructive stones in the CBD. An anatomical variation was noted, with the right posterior sectoral duct draining into the LHD and the right anterior sectoral duct draining into the CHD. Given the imaging findings, the surgeon expressed concern regarding a possible cholecystobiliary fistula.

**Figure 3:**
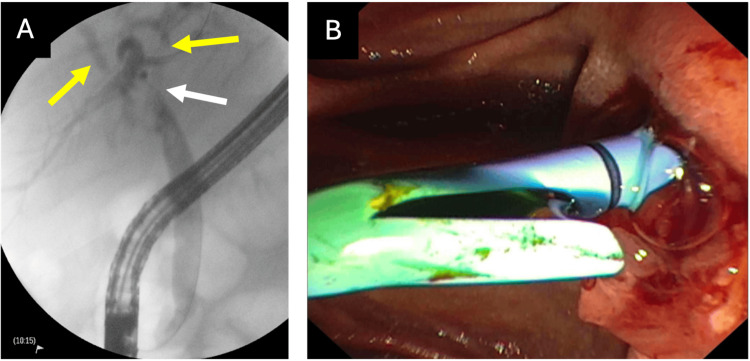
Preoperative endoscopic retrograde cholangiopancreatography (ERCP) findings. (A) ERCP imaging identified narrowing at the level of the hepatic duct confluence (white arrow) with moderate dilatation of the intrahepatic hepatic ducts (yellow arrows) without clear intraductal stones. (B) One 7 Fr 15 cm plastic stent was advanced into the left hepatic duct past the narrowing.

ERCP was performed to further define the anatomy and allow the placement of a plastic stent. The lower third of the CBD contained multiple stones (without complete obstruction). These were cleared. The left and right main hepatic ducts were moderately dilated due to compression at the bifurcation (Figure 3). However, no intraductal stones were identified. Two plastic stents were placed: one in the CBD and the other in the intrahepatic portion of the LHD. The endoscopist confirmed the diagnosis of MS but was unable to confidently diagnose a fistula. Follow-up CT confirmed well-placed stents and decreased intrahepatic duct prominence.

Two months after ERCP, the patient reported new symptoms, including generalized abdominal discomfort, poor appetite, and a general feeling of being unwell. After initial reservations about surgery, given his new symptoms, the patient consented to a laparoscopic (total or subtotal) cholecystectomy and stone removal, with a low threshold for conversion to open surgery.

**Figure 4:**
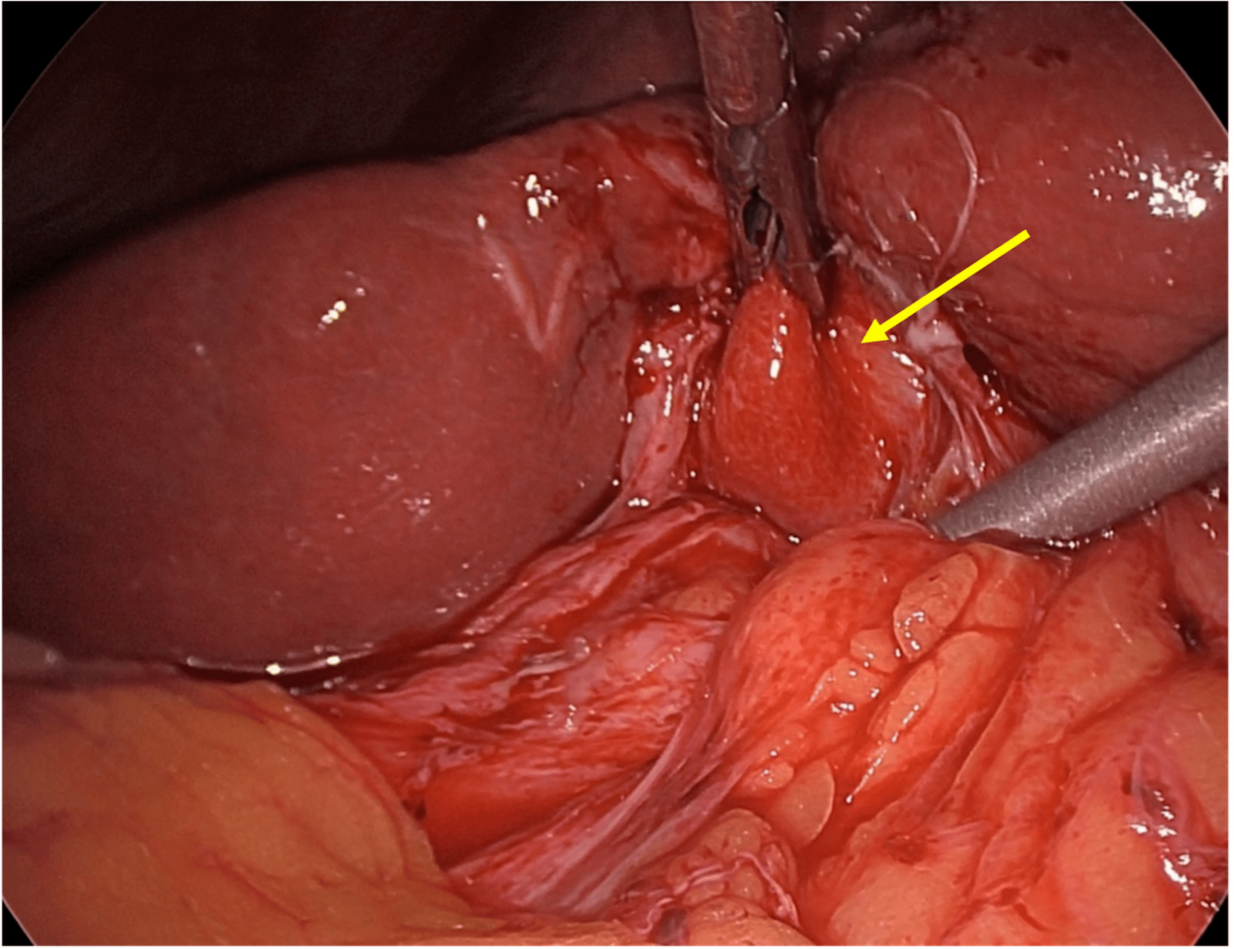
Laparoscopic findings. Laparoscopic view demonstrating severe chronic cholecystitis with dense adhesions to surrounding tissue and a deeply intrahepatic gallbladder (arrow). These findings, i.e., obliteration of normal tissue planes and inflammatory adhesion to adjacent structures, are characteristic of advanced Mirizzi syndrome and preclude standard anterograde cholecystectomy. In this case, a fundus-first approach was required.

The patient was admitted for elective surgery. The Hasson technique was used to establish pneumoperitoneum via a 10 mm infraumbilical port, followed by a 10 mm epigastric port and two right-sided 5 mm ports. Operative findings revealed severe chronic cholecystitis with markedly distorted anatomy. The gallbladder and adjacent liver were puckered and densely adherent (Figure 4).

**Figure 5:**
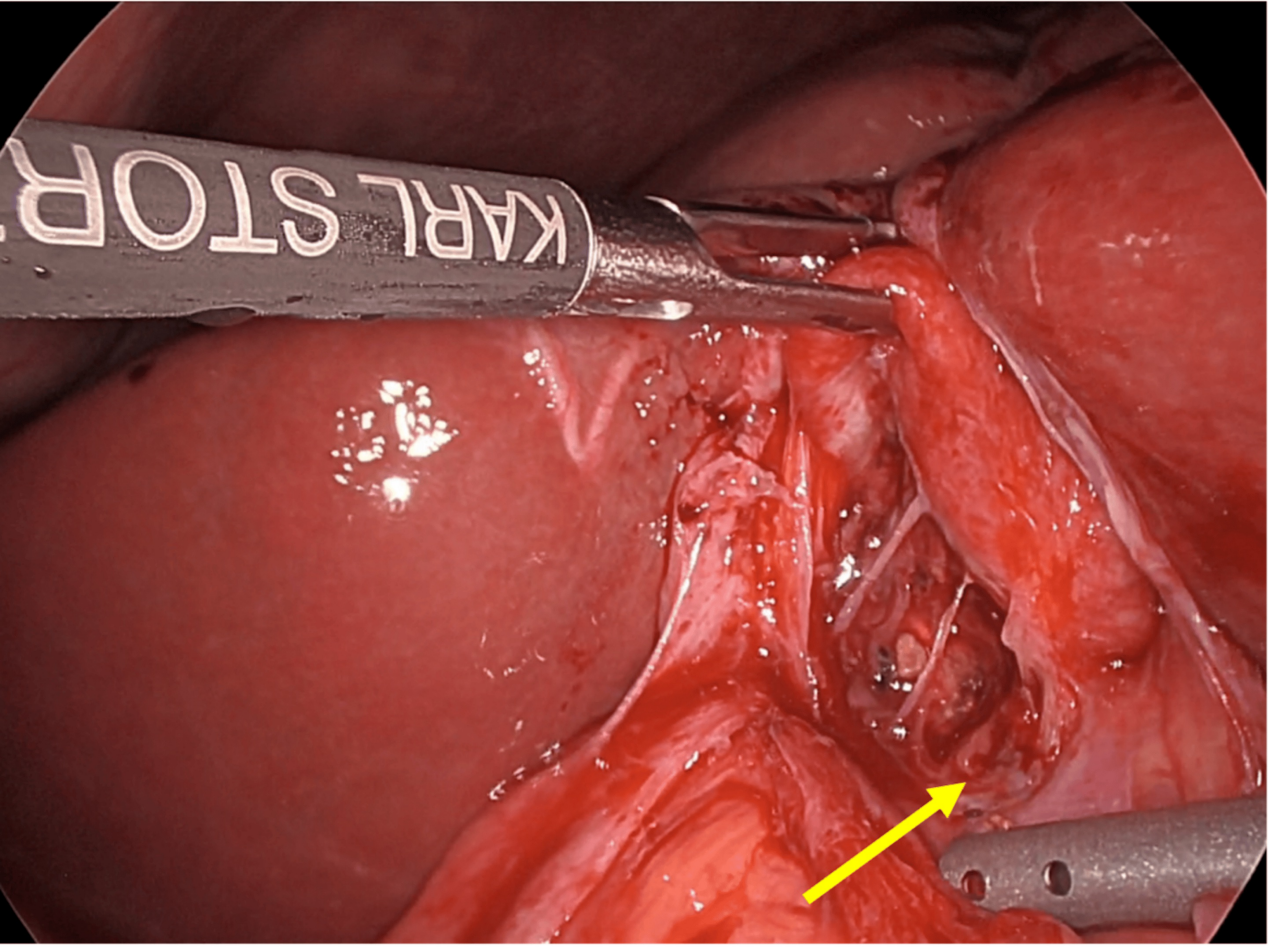
Laparoscopic photograph demonstrating an opened fundus with a bivalved gallbladder. Laparoscopic photograph demonstrating an opened fundus with a bivalved gallbladder. The peritoneal surface of the gallbladder remained densely adherent to the duodenum (arrow) and was left in situ to avoid inadvertent enterotomy. This technique, i.e., fenestrated subtotal cholecystectomy, prioritizes safety over complete gallbladder removal in the setting of hostile inflammation.

The gallbladder was deeply intrahepatic and inseparable from the duodenum because of dense adhesions. A fundus-first approach was used. The gallbladder was opened and bivalved, leaving the peritoneal surface of the gallbladder adherent to the duodenum (Figure 5). The fundus was then resected.

**Figure 6:**
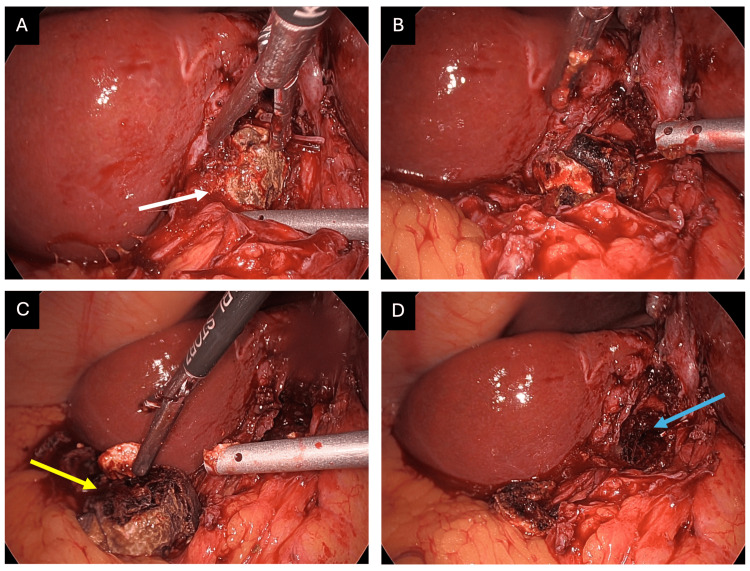
Removal of an eroding gallstone at the hepatic duct confluence. (A) A large, densely adhered gallstone (white arrow) is visible impacted at the hepatic duct confluence. (B) The stone was removed using controlled crushing and hydrodissection to minimize trauma to the surrounding bile duct walls. (C) The size of the extracted stone (yellow arrow) is evident in relation to the surrounding anatomy. (D) Significant mucosal erosion (blue arrow) is visible after stone removal, confirming a longstanding cholecystobiliary fistula. This degree of erosion illustrates why preoperative imaging may underestimate disease severity, chronic inflammation and fistulation are often only fully appreciated intraoperatively.

A large gallstone had eroded into the hepatic duct confluence and was densely adherent to the surrounding walls of the biliary system. The stone was partially fragmented and removed using a combination of hydrodissection and careful monopolar diathermy (Figure 6).

**Figure 7:**
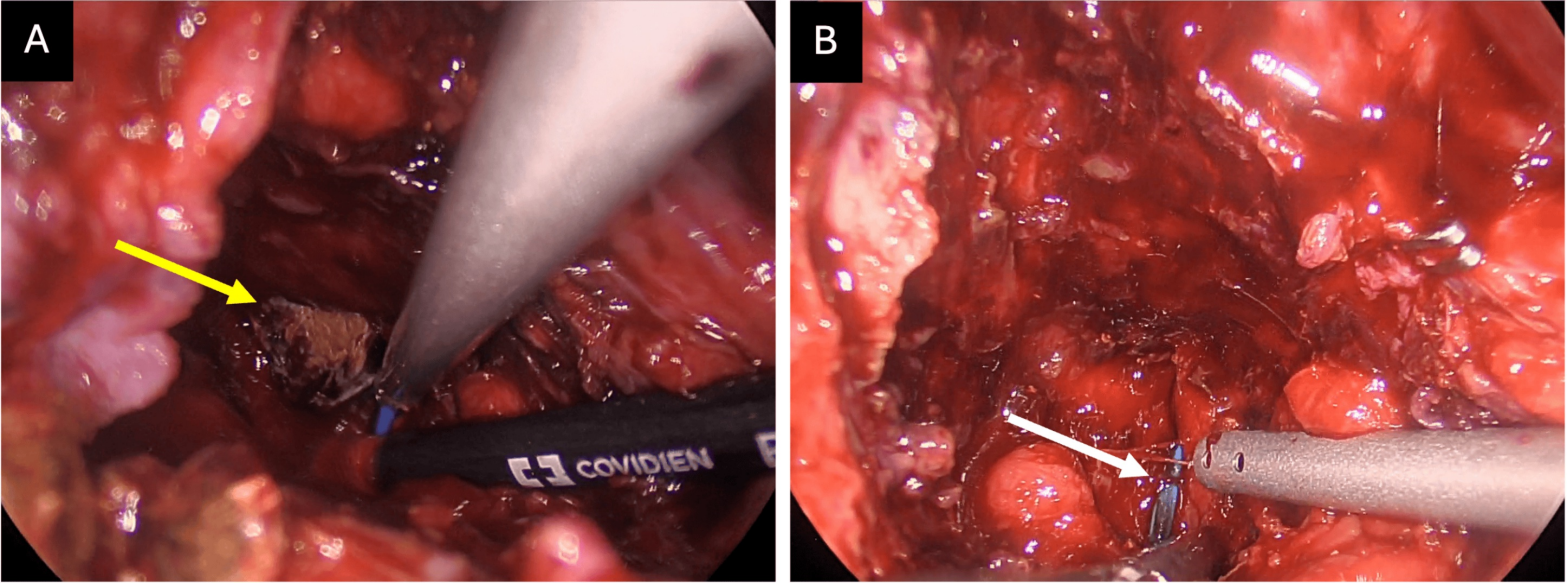
Confirmation of an intraductal left hepatic duct calculus. (A) The most proximal calculus seen on preoperative imaging (yellow arrow) was identified and removed from within the hepatic duct confluence. (B) After stone removal, the left hepatic duct (LHD) biliary stent placed during preoperative endoscopic retrograde cholangiopancreatography was clearly visible (white arrow). This confirmed that the stone was truly intraductal, having migrated through a cholecystobiliary fistula into the LHD, rather than causing extrinsic compression as suggested by magnetic resonance cholangiopancreatography. The stent served as a useful intraoperative landmark and facilitated identification of the biliary lumen.

Once the most proximal stone identified on preoperative imaging was removed, the endoscopically placed biliary stent was clearly visible within the LHD (Figure 7), confirming a cholecystobiliary fistula.

Taken together, the operative findings were consistent with MS complicated by a sizeable cholecystobiliary fistula, in contrast to the MRCP impression of extrinsic compression by extruded stones from a chronically perforated gallbladder. Notably, these fistulous changes were not definitively demonstrated on ERCP.

The cystic artery was identified and clipped flush with the right hepatic artery because of the distorted anatomy. No bile leakage was observed at the time of stone removal. Preoperative MRCP and CT images were reviewed intraoperatively to guide the dissection. Placement of a T-tube was considered but ultimately avoided to prevent interference with the pre-existing biliary stents and their planned endoscopic removal. Given the patient’s significant cardiometabolic comorbidities and the hostile operative field, the decision was made to accept controlled external drainage via a subhepatic drain rather than attempt immediate biliary reconstruction, which would have required more extensive dissection and prolonged operative time. The presence of well-positioned biliary stents was expected to maintain antegrade drainage to the ampulla and mitigate the impact of any bile leak from the fistula site.

A 15-Fr Blake subhepatic drain was inserted and positioned at the site of the final stone extraction to provide controlled external drainage in the presence of a large cholecystobiliary defect. The shrunken, bivalved gallbladder remnant effectively sealed around the drain exit site. After copious irrigation and confirmation of haemostasis, the abdomen was desufflated, port sites were closed, and the drain was secured.

Overall, the procedure constituted a laparoscopic fenestrated subtotal cholecystectomy with removal of large gallstones that had eroded into the biliary tree.

The immediate postoperative course was complicated. The patient developed a severe acute gout flare, resulting in significant pain and limited mobility. A CT scan performed two weeks postoperatively demonstrated a new subcapsular hepatic lesion in segment VI, consistent with an evolving abscess. Following consultation with the infectious diseases team, prolonged antibiotic therapy was initiated. The patient was discharged with his drain left in situ and plans for gradual shortening and eventual removal.

At one month postoperatively, the patient presented with worsening abdominal pain and fevers after accidental drain dislodgement. CT imaging demonstrated enlargement and multiloculation of the hepatic abscess. USS-guided percutaneous drainage was performed, resulting in significant symptomatic improvement. Repeat ERCP several days later confirmed no retained intraductal stones, and the biliary stents were removed. The patient’s condition improved progressively, and follow-up USS at three months confirmed resolution of the hepatic abscess.

**Figure 8:**
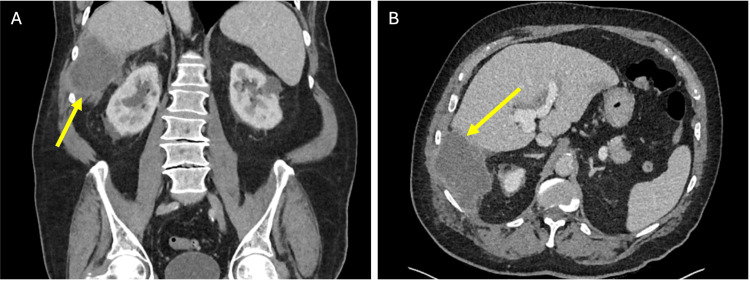
Computed tomography (CT) scan on presentation to the emergency department six months postoperatively with abdominal pain and fevers. Portal venous phase CT demonstrates a new perihepatic abscess (yellow arrows) in coronal (A) and axial (B) views.

Almost six months later, the patient presented to the emergency department with right flank pain and fevers. Blood tests revealed elevated inflammatory markers and mildly elevated cholestatic enzymes. CT and USS imaging revealed a large perihepatic subphrenic abscess containing debris and adjacent stones (Figure 8).

**Figure 9:**
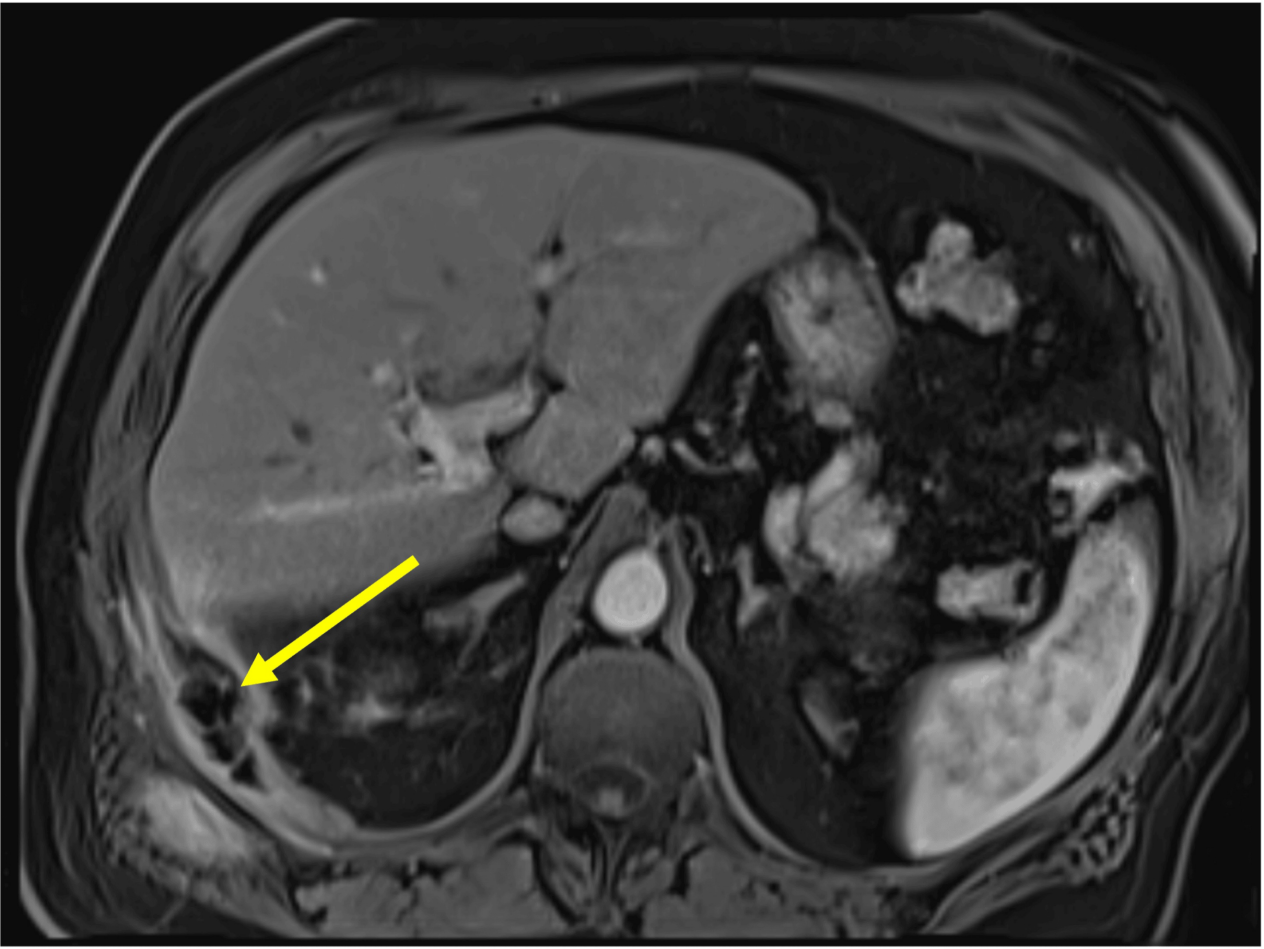
Magnetic resonance cholangiopancreatography (MRCP) findings 10 days after image-guided drainage of the perihepatic abscess. Axial T2-weighted HASTE sequence demonstrates multiple calculi (yellow arrow) up to 16 mm in size within the perihepatic abscess cavity.

An image-guided 12-Fr locking-loop drain was inserted. MRCP performed 10 days later demonstrated five to six retained stones measuring up to 16 mm within the abscess cavity (Figure 9).

**Figure 10:**
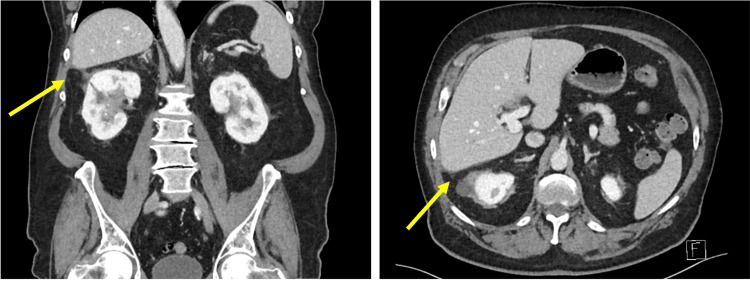
Computed tomography (CT) scan performed after removal of the surgical drain within the perihepatic collection. Portal venous phase CT images demonstrated complete resolution of the previous perihepatic collection (yellow arrows) in coronal (A) and axial (B) views.

Following appropriate antibiotic therapy and confirmation of adequate drainage on repeat USS, the patient returned to theatre for a laparoscopic adhesiolysis, washout of the subhepatic abscess cavity, and retrieval of retained gallstones. Multiple stones inferior to the right lateral liver edge were identified and retrieved. The previous percutaneous drain was removed, and a new surgical drain was placed in the subhepatic space. At review one month later, the patient had recovered well. CT imaging after removal of the surgical drain confirmed complete resolution of the perihepatic collection (Figure 10). At the final follow-up, approximately nine months after the index operation, the patient remained asymptomatic with unremarkable liver function tests and had returned to his baseline functional status. No further biliary interventions were required.

## Discussion

Advanced fistulating MS is infrequent within the MS, and hilar confluence or hepatic duct involvement has only been sporadically described [1,3,5,8]. We were unable to identify prior reports describing gallstones migrating through a cholecystobiliary fistula across the hepatic duct confluence to lie within the LHD in the setting of pre-existing variant biliary anatomy. Therefore, this case represents a rarely reported configuration within the Mirizzi spectrum.

Beyond its anatomical rarity, this case highlights that advanced fistulating MS may present with a deceptively subtle clinical profile. Marked cholestatic biochemistry with preserved bilirubin levels and minimal symptoms should raise suspicion for partial or intermittent biliary obstruction. In this patient, the imaging findings that warranted further scrutiny included loss of the fat plane between the gallbladder and hepatic hilum, apparent extrinsic biliary compression at or above the confluence, and variant biliary anatomy. The discordance between non-invasive imaging and ERCP findings in this case underscores the importance of anticipating more advanced disease than preoperative studies may suggest.

An important challenge in MS is accurate preoperative characterization. Preoperatively unrecognized MS is associated with higher bile duct injury rates, increased conversion to open surgery, and postoperative morbidity [1,18]. No single modality is reliably effective. Ultrasonography, although an appropriate first-line imaging modality, has the poorest reported performance in MS, with a sensitivity of 8%-57% and diagnostic accuracy of approximately 29% across series [1,9,10]. CT is useful for excluding malignancy and demonstrating gallbladder wall thickening, lymphadenopathy, and extraluminal processes; however, the findings are typically non-specific for MS [1,2,9]. MRCP is preferred for non-invasive biliary mapping and has reported sensitivity of 77%-100% for MS. However, its efficacy declines significantly in the diagnosis of cholecystobiliary fistulas [1,8,9]. This limitation was evident in our case: MRCP supported the diagnosis of MS and identified the anatomical variant, yet the proximal stones were interpreted as extrinsic compression rather than intraductal stones communicating via a fistula.

ERCP complements MRCP by providing definitive cholangiography and therapeutic decompression. Contemporary studies report high sensitivity (up to 100%) and diagnostic accuracy (up to 90%) for ERCP in MS, while also enabling sphincterotomy, stone extraction, and stent placement [1,10,11]. Importantly, ERCP is not without risk. Complications include pancreatitis (approximately 3.5%), hemorrhage (1.3%), cholangitis (1%), and perforation (0.1%-0.6%) [1,10]. In our patient, preoperative ERCP achieved distal duct clearance and stenting. Intraoperatively, the stent position provided a practical landmark and helped confirm that the proximal stones were intraductal at the confluence and LHD.

Pre-existing biliary anatomical variations further increase diagnostic and operative complexity. Variants are observed in approximately 20% of individuals, and sectoral ducts exhibiting atypical drainage patterns may impact the distribution of obstructions and elevate the risks associated with incorrect identification of ductal anatomy [1,2]. In this case, the right posterior sectoral duct drained into the LHD and the right anterior sectoral duct drained into the CHD, plausibly influencing both the stone distribution at the hilum and the risk profile of any dissection around the confluence.

Therefore, the operative strategy must be individualized according to disease extent, tissue quality, anatomy, and physiological reserve. Historically, open surgery has been the predominant approach because Calot’s triangle is frequently obliterated in MS. Early laparoscopic series reported high conversion (up to 65%) and bile duct injury rates of up to 14%-30% [12,13,18,19]. Over the past decade, however, specialist-unit series and systematic reviews have increasingly supported laparoscopic management with the incorporation of risk-mitigating techniques (such as fundus-first dissection and subtotal cholecystectomy) and maintenance of a low threshold for conversion to open surgery [4,12-17,20]. In this patient, severe chronic inflammation with complex fistulation, along with significant cardiometabolic comorbidities, made prolonged open reconstruction less favorable. A laparoscopic fundus-first approach with fenestrated subtotal cholecystectomy allowed stone clearance while limiting dissection in a hostile Calot’s triangle. The presence of well-positioned biliary stents likely promoted antegrade drainage via the ampulla and reduced the impact of any anticipated bile leak, supporting a strategy of controlled drainage rather than immediate ductal reconstruction [1,8,10,20].

## Conclusions

This case describes a complex fistulating MS with intraductal stones at the hepatic duct confluence and LHD, occurring in the context of pre-existing variant biliary anatomy. Despite preoperative MRCP and ERCP, the intraductal location of the proximal stones and the extent of fistulation were only confirmed intraoperatively. In high-risk patients with hostile Calot’s anatomy, a laparoscopic fundus-first approach with fenestrated subtotal cholecystectomy and controlled drainage can achieve ductal clearance while prioritising the avoidance of major bile duct injury. However, this approach is situational, and its feasibility depends on patient-specific factors, including physiological reserve, the availability of pre-placed biliary stents to maintain antegrade drainage, and institutional expertise in complex hepatobiliary surgery. Clinicians pursuing this strategy should anticipate the risk of retained stones and infective sequelae and ensure access to image-guided drainage, ERCP, and staged reintervention when required.
